# Supplementary material for: Effects of Albumin on Survival after a Hepatic Encephalopathy Episode: Randomized Double-Blind Trial and Meta-Analysis
Source: J Clin Med. 2021 Oct 23;10(21):4885. doi: 10.3390/jcm10214885 (PMC8584661; doi:10.3390/jcm10214885)
Supplement: Supplementary file 1 [file jcm-10-04885-s001.zip › jcm-1421104-supplementary.pdf]

Journal of
Clinical Medicine

MDPI

Supplementary online material

Supplementary methods

ALFAE inclusion and exclusion criteria Inclusion criteria were: liver cirrhosis; development of an episode of HE that was initiated within 72 h of inclusion into the study and persisted on grade 2 (West-Haven scale); age between 18 and 85 years and a signed consent. Exclusion were: terminal illness, need for intensive support (ventilation, dialysis, vasopressors, etc.); psychiatric or neurological conditions that make the assessment of HE difficult; disorders requiring treatment with albumin, contraindications to albumin, active gastrointestinal bleeding in the previous 72 h, acute-on-chronic liver failure (ACLF) defined by an acute decompensation associated with bilirubin >5 mg/dl.

Visits protocol All the visits included a physical exam with evaluation of overt HE (West Haven and Clinical hepatic encephalopathy staging CHESS scales,) and minimal hepatic encephalopathy (MHE), laboratory tests and evaluation of adverse events (AEs) (causal relation with the study medication was). MHE was assessed with the Psychometric hepatic encephalopathy score (PHES). Patients were followed during a 6-month period or until dead or receiving a liver transplant with monthly visits during the first three months and telephonic controls thereafter. The last visit was performed at day 180.

Table S1. List of participant centres.

Center	City
Hospital Universitari de la Vall d'Hebron	Barcelona
Hospital Universitari de la Santa Creu i Sant Pau	Barcelona
Hospital Germans Tries i Pujol	Badalona
Hospital Universitari Parc Taulí	Parc Taulí
Hospital del Mar	Barcelona
Hospital Broggi	Sant Joan d'Espí
Hospital Clínic	Barcelona
Hospital Gregorio Marañon	Madrid
Hospital Puerta del Hierro	Madrid
Hospital Marquez de Valdecillas	Santander
Hospital Universitario Ramón y Cajal	Madrid
Hospital Universitari Bellvitge	Barcelona
Hospital de Terrassa	Terrassa
Hospital Virgen del Rocío	Sevilla

Table S2. Baseline characteristics of ALFAE and BETA studies.

	BETA Placebo group	BETA Albumin group	ALFAE Placebo group	ALFAE Albumin group	P between all ALFAE and BETA studies	P Between both Placebo groups	P Between both Albumin groups
	n = 42	*n* = 40	*n* = 30	*n* = 26			
**Male**, *n* (%)	26(61.9)	29(72.5)	23(76.7)	19(73.1)	0.5	1	0.2
**Age**, median (IQR)	69.1(63.3–75.3)	66.5(59.9–73.6)	66(61–72)	62(56–73)	0.3	0.5	0.4

**Ideal weight**, median (IQR)	65.9 (61–79.2)	63 (55–68)	69.2 (62.3–73.5)	65 (62–70)	0.2	0.053	0.3
**Etiology**, *n*(%)							
Alcohol related	22(52.3)	19(47.5)	17(56.7)	7(26.9)	0.5	0.8	0.1
MAFL	2(4.8)	4(10)	--	--	0.08	0.5	0.1
Alcohol and HCV	4(9.5)	6(15)	2(6.7)	5(19.2)	1	1	0.7
HCV	5(11.9)	6(15)	10(33.3)	8(30.7)	0.01	0.04	0.1
Alcohol and MAFL	2(4.8)	--	--	--	0.5	0.5	--
Other *	7(16.6)	5(12.5)	1	6	0.6	0.1	0.7
**Previous decompensations**							
Ascites	35(83.3)	30(75)	27 (90)	20 (76.9)	0.5	0.5	1
Hepatic encephalopathy	27(64.3)	26(65)	22(73.3)	14 (53.8)	1	0.5	0.4
Spontaneous bacterial peritonitis	7(16.7)	7(17.5)	9 (30.0)	4 (15.4)	0.4	0.3	1
Gastrointestinal bleeding	11(26.2)	11(27.6)	11 (36.7)	5 (19.2)	0.8	0.4	0.6
Hepatorenal syndrome	2(4.8)	3(7.5)	1 (3.3)	2 (7.7)	1	1	1
Hepatocellular carcinoma	5(11.9)	4(10)	6(20)	3 (11.5)	0.6	0.7	1
**Precipitating Factors** **							
Infections, *n* (%)	11(26.1)	12(30)	14(53.8)	11(36.7)	0.048	0.08	0.4
Constipation, *n* (%)	9(21.4)	9(22.5)	12(30)	10(33.3)	0.6	1	0.6
Dehydration, *n* (%)	4(9.5)	3(7.7)	8(30.8)	10(33.3)	0.002	0.01	0.003
Diuretic, *n* (%)	14(33.3)	23(59)**	8(38.5)	10(53.3)	0.2	0.6	0.2
**Laboratory parameters,** median (IQR)							
Hemoglobin g/dL	10.6(9.9–13)	10.9(9.3–11.8)	11(9.6–13.4)	10.7(9.7–12.3)	1	0.7	0.9
Leukocytes x109/L	5.06(3.8–7.4)	5.09(4.06–6.6)	5.3(4.4–6.8)	5.3(4.4–7.9)	0.8	0.5	0.5
Platelets x10E9/L	82.5(64–109)	76.5(57.5–106.5)	105(80–134.7)	87.7(67.8–125)	0.03	0.05	0.2
Sodium mEq/L	135.8(132.8–138.6)	136.2(134–139)	134.5(130–139)	134.5(126–140)	0.08	0.3	0.2
AST IU/L	44(31–64)	51(36–75)	40(26–54)	45.5(29–81.7)	0.2	0.5	0.3
ALT IU/L	26(18–35)	32.5(22–43.5)	23.5(15.3–35.5)	26.5(20–47.5)	0.2	0.5	0.4
Bilirubin mg/dL	3.2(1.7–4.6)	2.97(1.91–5)	2.1(1.3–2.9)	2.7(1.9–3.8)	0.018	0.014	0.3
Albumin g/dL	2.85(2.35–3.01)	2.6(2.41–2.93)	3.1(2.5–3.4)	2.85(2.4–3.3)	0.2	0.2	6
INR	1.53(1.37–1.71)	1.55(1.35–1.83)	1.43(1.32–1.64)	1.6(1.37–1.78)	0.3	0.2	0.9
Creatinine (mg/dL)	1.08(0.77–1.53)	0.99(0.7–1.32)	1.38(0.89–1.6)	0.87(0.68–1.11)	0.9	0.4	0.4
MELD score	17 (16–20)	17(15–20)	15(11.8–20)	17(15–19)	0.2	0.04	0.3
Child-Pugh score	10(8–11)	10(8–11)	9(7–10)	8.5(7–10)	0.02	0.05	0.02

** In 3 patients from the ALFAE study, precipitating factors could not be identified. * Wilson, double viral etiology (HCV &HBV), triple etiology (HCV, HBV and OH), hemochromatosis and alfa-1 antitrypsin.

Table S3. Non-severe adverse events presented during the study.

**Organ system preferred term**	**Albumin *** *n* **= 40 patients**	**Placebo **** *n* **= 41 patients**

**Investigators**		
Hyperkalemia	1	1
Hyponatremia	4	2
Hyperbilirubinemia	1	2
Thrombocytopenia	--	3
**Gastrointestinal disorders**		
Abdominal pain	2	5
Diarrhea	4	1
Constipation	1	1
Nausea-Vomiting	--	3
GI bleeding	2	
Hernia	--	1
Colitis	--	
Gallbladder or choledochal stones	--	1
Pancreatitis	1	
**Metabolism and nutrition disorders**		
Hyperglycemia	2	3
Hypothyroidism	--	1
Nutrition disorder	1	--
**Blood and lymphatic system disorders**		
Anemia	14	9
**Nervous system disorders**		
Seizures	1	--
Headache	1	2
Paresthesia	1	--
Gait disturbance	1	--
Cerebral ischemic disease	--	2
**Injury, poisoning and procedural complications**		
Bone fractures and traumatisms *	5	3
**Vascular disorders**		
Hypertension	1	3
Hypotension	2	2
Hematoma	--	1
**General disorders and administration-site conditions**		
General physical deterioration-unspecific pain	3	--
Asthenia	--	2
**Cirrhosis related ***		
Ascites	14	24
Oedemas	4	6
Hepatic encephalopathy	11	8
Spontaneous Bacterial Peritonitis♦	1	1
Hepatorenal syndrome	--	1
Portal vein thrombosis	1	--
**Infections and infestations**		
Genitourinary	6	7
Localized fungal infection	2•	1
Respiratory (bacterial or viral)	4	1
Cellulitis or cutaneous infection	2	--

Colonization by resistant bacteria	1	--
Bacteraemia	3	1
Phlebitis	2	2
Gastroenteritis (viral)	1	--
Conjunctivitis	--	1
**Renal and urinary disorders**		
Hematuria	--	2
Acute kidney injury	10	2
**Neoplasms benign, malignant and unspecified**		
Benign	--	1
HCC novo or progression	1	2
**Respiratory,thoracic and mediastinal disorders**		
Bronchospasm	1	--
Pleural effusion	1	--
Pulmonary Hypertension	1	--
Dyspnoea	--	2
Respiratory failure	2	4
**Psyquiatric disorders**		
Insomnia	1	--
Depression	--	1
Confusion	--	1
**Skin and subcutaneous tissue disorders**		
Pruritus	1	1
Psoriasis	1	--
Rash	--	1
**Cardiac disorders**		
Aortic valve disease	1	--
Heart failure	3	--
Bradycardia	--	1

* In the albumin group there were also one episode of: back pain, conjunctival hemorrhage, cough, electrolyte imbalance, skin neoplasm excision, muscle spasm, thermal burn, tongue ulceration, anal fissure, alcohol related liver decompensation, tremor and treponema test positive. ♦ Including one bacterioascites. • vaginal and cutaneous. ** In the placebo group there were also one episode of anorexia, diabetic neuropathy, dizziness, dyslipidemia, unfocalized pain, perianal erythema, peritoneal hemorrhage, portal shunt, sciatica, syncope, diabetes, agitation, deep vein thrombosis, pyrosis, unspecific chest pain, coagulation worsening and urinary retention. None of the adverse events were considered to be related to the study medication.

Table S4. Adverse events that were considered as severe at least once during the study.

Organ system preferred term	Albumin *n* = 20 with at least one SAE Recovery or ongoing/dead	Placebo *n* = 23 with at least one SAE Recovery or ongoing/dead
**Investigators**		
Hyponatremia	1/0	--
Hyperbilirubinemia	--	1/0
**Gastrointestinal disorders**		
GI bleeding	--	3/0
Hernia	1/0	1/0
Ischemic colitis	--	0/1

Gallbladder or choledochal stones	1/0	1/0
Necrotizing esophagitis	--	0/1
**Blood and lymphatic system disorders**		
Anemia	5/0	2/0
**Injury, poisoning and procedural complications**		
Bone fractures and traumatisms	2/0	1/0
**General disorders and administration-site conditions**		
Fatigue	2/0	--
Multi-organ organ failure	0/1	0/1
Malaise	--	1/1
**Cirrhosis related**		
Ascites	2/0	1/0
Hepatic encephalopathy	8/2	9/2
Spontaneous Bacterial Peritonitis	1/0	1/1
Hepatorenal syndrome	1/3	1/0
Acute-on-chronic liver failure	0/1	0/1
Slow portal blood flow	1/0	--
**Infections and infestations**		
Genitourinary	1/0	2/1
Respiratory	2/0	1/0
Cellulitis	1/0	1/0
Bacteraemia	1/0	0/1
Endocarditis	0/1	--
Cholecystitis	1/0	1/0
Septic shock	--	1/2
**Renal and urinary disorders**		
Acute kidney injury	0/1	1/4
**Neoplasms benign, malignant and unspecified**		
Basal cell carcinoma	1/0	--
HCC novo or progression	3/0	--
Lung neoplasm	1/0	--
**Respiratory, thoracic and mediastinal disorders**		
Pulmonary Hypertension	--	1/0
Aspiration	--	1/0
Pulmonary edema	1/0	--
Respiratory failure	--	0/1
**Cardiac disorders**		
Bradycardia	1/0	--

None of the adverse events were considered to be related to the study medication. Severe adverse event, SAE; Gastrointestinal, GI; hepatocellular carcinoma, HCC.

Table S5. Time frame to death in both clinical trials.

EXITUS date	BETA albumin *n* = 40	BETA placebo *n* = 42	ALFAE treatment *n* = 26	ALFAE Placebo *n* = 30
**0–7 days**	--	1	1	--
**7–15 days**	--	1	--	1
**15–28 days**	--	2	0	3
**29–60 days**	2	4	3	2

**61–90 days**	1	--	2	8
**91–180 days**	2	3	--	--

Table S6. Time frame to liver transplant.

**Transplant date**	**BETA albumin** *n* = 40	**BETA placebo** *n* = 42	**ALFAE treatment** *n* = 26	**ALFAE Placebo** *n* = 30
**0–7 days**	--	--	--	--
**7–15 days**	1	--	--	--
**15–28 days**	1	--	--	1
**29–60 days**	--	--	--	1
**61–90 days**	--	--	1	2
**91–180 days**	1	2	--	--

Table S7. 90-days mortality risk factors for both cohorts.

		**Multivariate Cox Regression**		
**VARIABLE**		**HR**	**(95% CI)**	***p*-value**
Treatment group	Placebo	1		0.004
	Albumin	0.18	(0.06; 0.57)	
Sodium		0.91	(0.85; 0.98)	0.012
MELD		1.13	(1.01; 1.27)	0.034
CHESS scale		1.31	(1.14; 1.52)	<0.001

Harrell C = 0.7981, AIC model=175.75.56, BIC model=186.59. Abbreviations Model for end stage of liver disease, MELD; Clinical hepatic encephalopathy staging scale, CHESS.

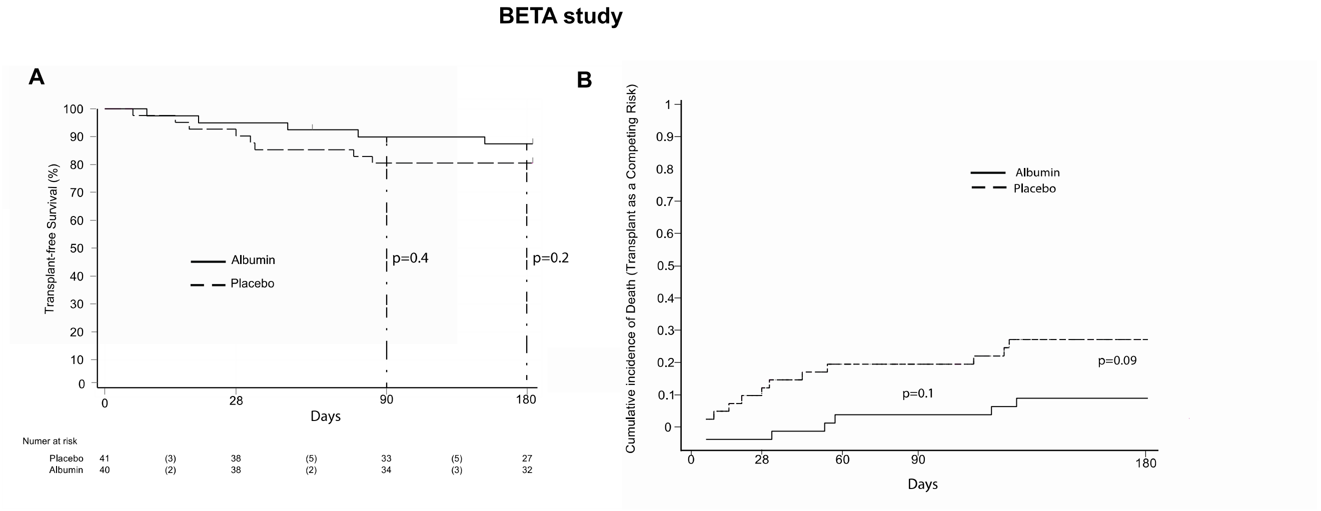

Figure S1. 180 days Transplant-free Survival and Cumulative incidence of Death with Transplant as a competing risk (**A**) Kaplan-Meier estimates of Transplant-free Survival at 180 days. (**B**) 180 days cumulative incidence of Death with Transplant as a competing risk
